# Questionnaire study suggests grave consequences of infectious laryngotracheitis, infectious coryza and mycoplasmosis in small chicken flocks

**DOI:** 10.1186/s13028-023-00703-z

**Published:** 2023-09-14

**Authors:** Pernille Engelsen Etterlin, Arianna Comin, Helena Eriksson, Elisabeth Bagge, Tomas Jinnerot, Liv Jonare, Désirée S. Jansson

**Affiliations:** 1 Department of Animal Health and Antimicrobial Strategies, National Veterinary Institute, 751 89 Uppsala, Sweden; 2 Department of Disease Control and Epidemiology, National Veterinary Institute, 751 89 Uppsala, Sweden; 3 Department of Microbiology, National Veterinary Institute, 751 89 Uppsala, Sweden; 4 Department of Clinical Sciences, Swedish University of Agricultural Sciences, Box 7054, 750 07 Uppsala, Sweden

**Keywords:** Backyard poultry, Hobby flocks, Infectious coryza, Infectious laryngotracheitis, Molecular diagnostics, Mycoplasmosis, Respiratory infection

## Abstract

**Background:**

A growing number of people in western countries keep small chicken flocks. In Sweden, respiratory disease is a common necropsy finding in chickens from such flocks. A respiratory real-time polymerase chain reaction (PCR) panel was applied to detect infectious laryngotracheitis virus (ILTV), *Avibacterium paragallinarum* (*A*. *paragallinarum*) and *Mycoplasma gallisepticum* (*M. gallisepticum*) in chickens from small flocks which underwent necropsy in 2017–2019 and had respiratory lesions. Owners (*N* = 100) of PCR-positive flocks were invited to reply to a web-based questionnaire about husbandry, outbreak characteristics and management.

**Results:**

Response rate was 61.0%. The flocks were from 18 out of Sweden’s 21 counties indicating that respiratory infections in small chicken flocks are geographically widespread in Sweden. Among participating flocks, 77.0% were coinfected by 2–3 pathogens; 91.8% tested positive for *A. paragallinarum*, 57.4% for *M*. *gallisepticum* and 50.8% for ILTV. Larger flock size and mixed-species flock structure were associated with PCR detection of *M*. *gallisepticum* (P = 0.00 and P = 0.02, respectively). Up to 50% mortality was reported by 63.9% of respondents. Euthanasia of some chickens was carried out in 86.9% of the flocks as a result of the outbreaks. Full clinical recovery was reported by 39.3% of owners suggesting chronic infection is a major challenge in infected flocks. Live birds had been introduced in many flocks prior to outbreaks, which suggested these as an important source of infection. Following the outbreaks, 36.1% replaced their flocks with new birds and 9.8% ceased keeping chickens.

**Conclusions:**

This study highlights the severity of respiratory outbreaks in small non-commercial chicken flocks and points to the need for more research and veterinary assistance to prevent and manage respiratory infections in small chicken flocks.

**Supplementary Information:**

The online version contains supplementary material available at 10.1186/s13028-023-00703-z.

## Background

Keeping chickens for hobby or small-scale commercial purposes, henceforth referred to as small chicken flocks (SCF), has become popular in European and North American countries [1–4]. Information on population size, structure, geographic location, animal husbandry, disease occurrence and disease management in Swedish SCF has remained limited while corresponding populations have received some attention in several other countries [3–7].

There are several reasons for keeping SCF, but some reports have shown that it is sometimes due to a wish of self-sufficiency of eggs and meat and/or a perception of a higher animal welfare level in SCF compared to large commercial farms [8, 9]. However, undiagnosed and untreated diseases may cause persistent flock problems and welfare issues and can be challenging for SCF owners [1]. There is also a potential risk of transmission of pathogens to commercial chickens [2, 8, 10]. Hence, better knowledge of diseases in SCF and how diseases are managed by SCF owners is essential.

Several studies have indicated that a variety of parasites and pathogenic organisms may be widespread among European and American SCF, some of which may cause respiratory infections [2, 3, 11, 12]. In recent years, respiratory signs have become one of the most common complaints of SCF owners when they submit chickens for necropsy to the National Veterinary Institute (SVA) in Sweden. SVA performs most of the laboratory poultry necropsies in Sweden [13] and is the only laboratory in Sweden providing molecular poultry diagnostics. Clinical signs, necropsy findings and results from molecular diagnostics performed at SVA suggest that infectious laryngotracheitis (ILT) caused by Gallid herpesvirus type 1 (GaHV-1)/infectious laryngotracheitis virus (ILTV) [14, 15], infectious coryza caused by *Avibacterium paragallinarum* (*A*. *paragallinarum*) [16, 17] and mycoplasmosis caused by *Mycoplasma gallisepticum* (*M. gallisepticum*) (synonym *Mycoplasmoides gallisepticum*) [18] are major causes of respiratory disease in SCF in Sweden. In contrast, these pathogens are very rarely diagnosed in commercial flocks in Sweden. This retrospective questionnaire study was designed to (1) record owners’ experience of clinical signs and clinical outcome of single or coinfections of ILTV, *A. paragallinarum* and *M*. *gallisepticum* in Swedish SCF and (2) gather information on how the submitting owners managed outbreaks of respiratory disease in their flocks.

## Methods

### Selection of flocks

All submissions from privately owned SCF with respiratory signs submitted to SVA during 2017–2019 were eligible for participation in this study. Additional inclusion criteria were evidence of respiratory lesions at necropsy as described in the literature [14, 15, 17, 18] and polymerase chain reaction (PCR) detection of ILTV, *M*. *gallisepticum* and/or *A. paragallinarum*. A diseased flock was defined as one in which three or more birds showed the same clinical signs of disease within the last month, regardless of the number of submitted birds.

### Necropsy

Poultry pathologists examined up to three birds from each submission according to a routine in-house necropsy protocol (SVA) based on the literature [19]. The protocol included gross and microscopic examinations. Selective sampling for microscopy targeting respiratory lesions was made at the discretion of the pathologist and included eye lid and nasal mucosa, trachea and lungs. Routine processing [formalin fixation, processing, paraffin embedment, sectioning, and staining with haematoxylin & eosin (HE) and additional stains when necessary] were performed.

### Sampling and real-time PCR analysis

Individual choanal and tracheal swabs were collected from each of the examined chickens at necropsy using sterile cotton swabs. From these birds, pooled choanal and tracheal swabs from the same birds were also collected. Samples were subsequently stored at −70 °C in 1.8 mL CryoTubeTM vials (Thermoscientific) for further PCR analysis for ILTV, *M*. *gallisepticum* and *A. paragallinarum* using primers and probes listed in Table 1. Swabs were vortexed in 850 µL TE buffer (10 mM Tris–HCl, 1 mM EDTA, pH 8.0) and nucleic acid was extracted from the liquid using either Bullet Stool kit (Hain Lifescience GmbH, Nehren, Germany) or IndiMag Pathogen kit (Indical Bioscience GmbH, Leipzig, Germany). Real-time PCR for *A. paragallinarum* and *M*. *gallisepticum* was carried out using previously published PCR assays [20, 21] respectively, with minor modifications. In short, the PCR for *M*. *gallisepticum* and *A. paragallinarum* was performed in separate reactions containing PerfeCTa qPCR ToughMix with Low ROX (Quantabio, Beverly, MA), 500 nM of each primer, 100 nM of probe and 2 µL sample extract at a total reaction volume of 15 µL. For detection of ILTV an in-house developed assay was used, and the PCRs consisted of KiCqStart One-Step Probe RT-qPCR ReadyMix with Low ROX (Merck KGaA, Darmstadt, Germany), 400 nM of each primer, 133 nM of probe and 2 µL sample extract at a total reaction volume of 15 µL. All PCR analyses were performed using an ABI 7500 Fast thermocycler (Life Technologies, Carlsbad, CA) and the following thermal profile: 50 °C for 10 min, 95 °C for 3 min and 45 cycles of 95 °C for 3 s and 60 °C for 30 s.

**Table 1:** Primers/probes used for PCR to detect *Avibacterium paragallinarum*, *Mycoplasma gallisepticum* and Gallid herpesvirus 1

Target organism	Oligo name	Sequence (5ʹ–3ʹ) with modifications	Refs
*A. paragallinarum*	APG-Qf	GCAAAAGACTACCAGCAAGGATAAT	20
	APG-Qr	CCTTACCCAAATATAATGTTCCACATT	
	APG-Pr	FAM-TCCTAGTTAGCATTATTGC-MGB	
*M. gallisepticum*	MG-Qf	GCTGGGTTGATTGTTGTTTCTT	21
	MG-Qr	TCTTCACGTTCTTGGATCATCAT	
	MG-Pr	FAM-CTCTTSGGTTTAGGGATTGGGATTCCG-IBFQ	
Gallid herpesvirus 1	IPC4 qPCR-F	CCCCACCCAGTAGAGGAC	In-house
	IPC4 qPCR-R	CGAGATACACGGAAGCTGATTT	
	IPC4-Pr	FAM-CAGTCTTTGGTCGATGACCCGC-TAMRA	

### Questionnaire survey

The owners of the submitted chickens who fulfilled the inclusion criteria were invited to participate in a questionnaire study. First, information about the study was sent by postal service. This was followed by a personal link to a web-based questionnaire created in Questback [22] and distributed by email. The questionnaire (translated from Swedish) is available as Additional file 1. Each participant received a unique identification code which enabled linking of laboratory results with survey answers. The survey included 23 questions, but as ten of these questions depended on a previous answer, the total number of questions varied between respondents. The questionnaire consisted of open and closed questions and multiple alternatives could be selected in some cases. An option for leaving a final open comment was also offered. The questions were categorized in five subsections; (1) background information, (2) flock status prior to the outbreak, (3) clinical course, (4) outbreak management and (5) post-outbreak management Additional file 1. All questions were mandatory, and the answers could only be submitted once. The survey was open from the 1st to the 31st of October 2020. A reminder was sent by email after 14 days and participants who could not be reached by email received the questionnaire by postal service. Non-responders were removed from the final data analysis.

### Data analysis

Data was initially summarised by means of descriptive statistics. Differences in the distribution of answers among respondents were assessed by means of chi-square test for each question. Statistical association between the occurrence of the three pathogens (i.e., ILTV, *M*. *gallisepticum* and *A. paragallinarum*) and potential risk factors at flock level were tested by means of Fisher’s test. P < 0.05 was considered statistically significant.

## Results

### Study flocks

A total of 529 chickens from 286 SCF were submitted to SVA during 2017–2019 for necropsy. One hundred and ninety-nine birds from a total of 100 flocks fulfilled the selection criteria (i.e., respiratory signs, respiratory lesions at necropsy and PCR detection of ILTV, *M*. *gallisepticum* and/or *A. paragallinarum*). The respiratory lesions detected at necropsy included serous to mucoid and/or haemorrhagic-necrotic conjunctivitis and/or sinusitis, laryngitis, tracheitis, airsacculitis and pneumonia. Out of the 100 flock owners, two could not be contacted and 61 completed the questionnaire.

### PCR analyses

Individual choanal slit and tracheal swab samples were available from 57 out of the 61 participating flocks. From the remaining four flocks, only pooled respiratory samples were available and used for PCR analysis. PCR results of the participating 61 flocks are presented at flock level, i.e., as summarised results of individual sampling of one to three birds or as pooled samples in Table 2. Among the participating flocks, 56 were infected with *A. paragallinarum* (91.8%), 31 with ILTV (50.8%) and 35 were *M*. *gallisepticum* positive (57.4%). Significantly more flocks were infected with *A. paragallinarum* (P < 0.00) than those that were not, whereas no difference in infection rates of ILTV and *M*. *gallisepticum* was noticed between the flocks.

**Table 2:** PCR detection of *Avibacterium paragallinarum*, *Mycoplasma gallisepticum* and Gallid herpesvirus 1 in 61 study flocks

PCR result	Number (%) of positive flocks
Gallid herpesvirus 1	2 (3.3)
*M. gallisepticum*	3 (4.9)
*A. paragallinarum*	9 (14.7)
Gallid herpesvirus 1 and *M. gallisepticum*	0
Gallid herpesvirus 1 and *A. paragallinarum*	15 (24.6)
*M. gallisepticum* and *A. paragallinarum*	18 (29.5)
Gallid herpesvirus 1*, M. gallisepticum* and *A. paragallinarum*	14 (23.0)

### Questionnaire survey

#### Background information

**Fig. 1:**
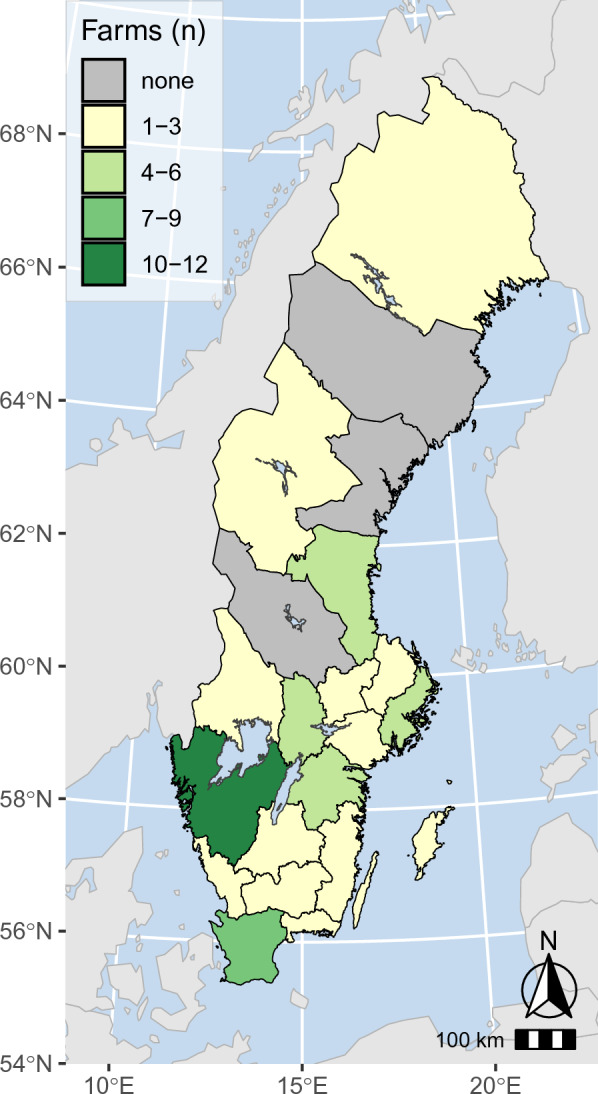
Chloropleth map showing the number and county location of small chicken flocks sampled in the present study

The 61 flocks were located in 54 out of 290 municipalities and in 18 out of Sweden’s 21 counties (Fig. 1). Most of the respondents came from southern Sweden. At the time of the survey, 55 respondents (90.2%) still kept chickens or other poultry. Eighteen out of the 55 respondents (37.2%) who kept chickens also kept at least one additional poultry species. These were kept either together with their chickens or as separate flocks and included ducks and/or geese in 14 flocks (25.5%), turkeys in four flocks (7.3%) and other poultry species in seven flocks (12.3%). Keeping other poultry species in addition to chickens was significantly associated with having a flock that was PCR-positive for *M*. *gallisepticum* (P = 0.02), whereas no such association existed for ILTV (P = 1.00) or *A. paragallinarum* (P = 0.16). Forty of the 55 owners who still kept chickens (72.3%) had flocks that consisted of more than one variety. Mixed breeds were found in 39 flocks (70.9%), pure-bred show breeds in 31 flocks (56.4%), heritage breeds in 32 flocks (58.2%), and layer hybrids were present in 14 flocks (25.5%). No broiler hybrids were reported by the respondents.

Most of the investigated flocks (*n* = 41; 67.2%) consisted of 11–50 birds, 9.8% (*n* = 6) had 51–100 chickens, 8.2% (*n* = 5) had 1–10 chickens and 5.5% (*n* = 3) had 101–250 chickens. Flock size and flock diversity were associated with each other i.e., larger flocks were more likely to include other poultry species in addition to chickens (P = 0.00). There was an association between larger flock size and *M*. *gallisepticum* PCR-positive result (P = 0.00). No association was shown between flock size and the detection of ILTV (P = 0.58) or *A. paragallinarum* (P = 1.00).

#### Flock status prior to the outbreak

Fifty-nine owners (96.7%) replied that their birds had not been vaccinated against any diseases within a year before the respiratory disease outbreak became evident and two did not know. Seven respondents (11.5%) had owned their chickens less than 6 months before the first clinical signs of respiratory disease were noticed. Fourteen respondents (23.0%) reported between 6–12 months ownership, 17 (27.8%) between 1–3 years ownership and 23 (37.7%) more than 3 years ownership before the first clinical signs of respiratory disease were noticed. There was a significant difference in how long the respondents had owned their flocks before the respiratory disease outbreak occurred (P = 0.03), but there was no association between ownership length and positive PCR results for ILTV (P = 0.22), *A. paragallinarum* (P = 0.92) or *M*. *gallisepticum* (P = 0.93).

Most owners (*n* = 50; 82.0%) had introduced new birds to their flock during the year before the outbreak (Table 3). Approximately one third (*n* = 7; 30.4%) of the 23 respondents who had owned their flock more than three years before respiratory signs appeared, had not introduced new birds during the preceding 12 months. Most owners recruited live birds instead of hatching eggs (Table 3). A significantly higher number (*n* = 52; 85.2%) of owners (P < 0.00) answered that they currently acquire birds from a source other than their own flocks (Table 3). The recruitment type (live birds or hatching eggs) was not associated with the PCR detection of ILTV (P = 0.84) *A. paragallinarum* (P = 0.42), *M*. *gallisepticum* (P = 0.14) or length of ownership.

**Table 3:** Summary of questionnaire results regarding flock characteristics in small chicken flocks

Characteristic	No. of flocks	%
Introduction of new birds/hatching eggs prior to outbreak (Q9)^a^		
No	10	16.4
Yes, within the recent month	19	31.2
Yes, within 1–5 months	20	32.8
Yes, within 6–12 months	11	18.0
I don’t recall	1	1.6
Introduction of live birds or hatching eggs (Q10)^b^		
Live birds only	33	66.0
Hatching eggs and live birds	10	20.0
Hatching eggs only	7	14.0
Source of new chickens (Q11)^c, d^		
No external source	9	16.7
From other small chicken farm	43	78.2
From commercial poultry farm	9	16.7
From live poultry markets/poultry shows	7	12.7
From abroad	0	0
The time of year the outbreak occurred (Q13)^a^		
Jan–Mar	4	6.6
Apr–Jun	13	21.3
Jul–Sep	11	18.0
Oct–Dec	13	21.3
I don´t know	20	32.8
Age groups which showed signs of disease (Q14)^a, d^		
Chicks (0 – 6 weeks)	9	14.8
Young birds (7 weeks – 5 months)	25	41.0
Adults (older than 5 months)	50	82.0
I don´t know	0	0
Clinical signs (Q15)^a, d^		
General signs of illness (less active, ruffled plumage)	43	70.5
Reduced appetite	18	29.5
Sneezing, coughing	48	78.7
Abnormal breathing sounds (rales, wheezing)	50	80.6
Blood or other discharge/secretions (on/in the beak, on the feathers or in the house/coop)	16	82.0
Swelling around the eyes or swollen head	33	54.1
Eye discharge	31	50.8
Decreased egg production	18	29.5
Mortality	42	68.2
Flock mortality during outbreak (% of flock) (Q16)^e^		
1–20	28	45.9
21–50	11	18.0
51–80 or 81–99 or all	0	0
I don´t recall	3	5.0
Euthanasia during outbreak (% of flock) (Q17)^a^		
None	7	11.5
1–20	26	42.6
21–50	3	5.0
51–80	1	1.6
81–99	0	0
All	23	37.7
I don´t recall	1	1.6

^a^All 61 owners received this question

^b^The 50 owners who had acquired new birds and/or hatching eggs prior to the outbreak received this question

^c^The 55 owners who still kept poultry after the outbreak received this question

^d^Multiple answers possible

^e^The 42 owners who had observed mortality during the outbreak received this question

#### Outbreak and post-outbreak management

Respiratory signs in the flocks occurred all year round (Table 3), but significantly fewer owners reported respiratory signs during the first 3 months of the year compared to other time periods (P = 0.00). There was a lower occurrence of *M*. *gallisepticum* PCR-positive flocks in January to March compared to the other months of the year (P = 0.03). No statistical association between the timing of the outbreaks and the PCR-positive ILTV (P = 0.38) or *A. paragallinarum* (P = 0.47) flocks was found. The owners reported a wide range of clinical signs in all age groups (chicks, young birds and adults) (Table 3). There was a significant association (P = 0.02) between young birds and *M*. *gallisepticum* PCR-positive flocks, whereas the other age groups (chicks and adults) had no association with *M*. *gallisepticum*. There was no association between age and ILTV or *A. paragallinarum* PCR-positive flocks. Mortality levels varied between 0–50% and euthanasia rates varied between 0–100% (Table 3). The number of owners who experienced mortality in their flocks (*n* = 42; 68.9%) or euthanized some or all of their chickens (*n* = 53; 86.9%) was significantly higher than those who didn’t (P = 0.00 and P < 0.00 for mortality and euthanasia respectively). There was no association between mortality and ILTV (P = 0.42), *A. paragallinarum* (P = 0.31) nor *M*. *gallisepticum* (P = 0.74).

Owners sought advice from both veterinarians (*n* = 54) and non-veterinarians (*n* = 24) on how to manage the outbreaks (Table 4). Treatment was reported by 6 out of 61 respondents (9.8%) (Table 4). Various combinations of supportive care (heat, massage, oral fluid replacement) and use of drugs such as anthelmintics (fenbendazole), acaricides (fluralaner), over-the-counter eye drops, mucolytica (bromhexine), non-steroidal anti-inflammatory drug (acetylsalicylic acid) or colloidal silver were reported. The number of flock owners who carried on keeping chickens after the outbreak (Table 4) was significantly (P < 0.00) higher than those who stopped. Full recovery (based on absence of clinical signs according to owners´ assessments) was reported by 39.3% (*n* = 24) of the respondents. Differences in hygiene (cleaning and disinfection) routines existed (Table 4). Three flock owners applied all hygiene measures presented in Table 4 and full recovery was reported in two of these flocks. Five of the eleven owners who didn’t perform any treatment, cleaning or disinfection also reported full flock recovery. No significant association was found between flock recovery and performance of none (P = 1.00) or all (P = 0.49) of the suggested hygiene measures (manure removal, soaking, wet cleaning, high pressure cleaning, disinfection) after the outbreak.

**Table 4:** Summary of questionnaire results regarding outbreak management in small chicken flocks

Characteristic	No of flocks	%
Source of advice (Q18)^a, b^		
Other poultry owners or non-professionals	24	39.3
Poultry veterinarians at SVA	52	85.2
Other veterinarians	21	34.4
Treatment (Q19)^a^		
None	55	90.2
Some birds were treated	2	3.3
All birds were treated	4	6.5
Flock destiny (Q22)^a^		
All birds were replaced with new ones	22	36.1
Some new birds were added to the flock	13	21.3
I kept my poultry, but I didn’t acquire more	20	32.8
I ceased keeping poultry	6	9.8
Cleaning and disinfection after the outbreak (Q23)^b, c^		
No cleaning/disinfection was carried out	11	20.0
Manure removal	27	49.1
Soaking	18	32.7
Wet cleaning	37	67.3
High pressure cleaning	15	27.3
Disinfection	30	54.5

Q numbers refer to question number in Additional file 1

^a^All 61 owners received this question

^b^Multiple answers possible

^c^The 55 owners who kept holding poultry received this question

## Discussion

This questionnaire-based study investigated the outcome and management of respiratory infections associated with ILTV, *A. paragallinarum* and *M*. *gallisepticum* alone or in combination in chickens from small non-commercial flocks with a laboratory confirmed post-mortem diagnosis. The outbreaks were often associated with severe clinical signs and high mortality and some owners euthanised a subset or all of their chickens. Information on the occurrence of respiratory pathogens in this poultry category in Sweden and elsewhere is scarce. At the time of this study, SCF were not registered in Sweden, and therefore the representativity of the results is unknown. However, the locations of the participating flocks in this study (Fig. 1) suggest that these infections are geographically widespread in Sweden. Several earlier studies from other countries suggest that respiratory infections are common in SCF both in Europe [2, 23] and North America [3, 4, 12]. In Finland, a neighbouring country to Sweden, 12% of 51 backyard chicken flocks were seropositive for ILTV [24]. Moreover, coinfections with several respiratory pathogens were common among the flocks in this study. This was also found in Canada and USA in SCF [3, 4]. Together, these results indicate that respiratory infection is an important issue in small non-commercial chicken flocks in several countries.

Response rates to questionnaires and interview surveys among small poultry flock owners varied widely (4–72%) in previously published studies [2, 11, 25, 26]. Other surveys have been advertised on internet platforms [1, 6, 8] where response rates could not be determined. Differences in response rates may be attributed to a variety of factors such as methodology, target population and incentives to respond. The response rate of this study (61.0%) was higher than in most previous reports. It is plausible that this was related to the combination of a postal informational letter, a known internet access (email addresses supplied at referral of necropsy cases) and an email reminder. Additionally, the questionnaire topic may have been of particular interest to the owners. When interpreting the results of this study, it must be remembered that it is likely to involve some degree of selection bias as the study specifically targeted owners who had submitted chickens for diagnostics and who could choose whether to participate. The severity of clinical signs and the mortality experienced by the responding owners may have been strong motives behind their decision to submit chickens for diagnostic necropsy and to participate in the questionnaire study. In an earlier study, 39% of small flock owners had chickens affected by respiratory disease within 1 to 6 months prior to the study, but only two reported mortalities [2]. This clearly shows that respiratory infections in SCF may not always be associated with the severe signs and mortality reported in our study.

In agreement with several earlier reports [1, 3, 6, 26], a mixed-species flock structure was a common trait in this study. This type of flock structure was associated with detection of *M*. *gallisepticum* by PCR, but not with *A. paragallinarum* or ILTV. *Mycoplasma gallisepticum* is known to infect a wide variety of poultry including gallinaceous and anseriform species [27], which could explain this association. In contrast, ILTV and *A. paragallinarum* both have a limited range of primary host species, i.e. mainly chickens [15, 17]. Further identified risks for *M*. *gallisepticum* detection in this study were age (young chickens having a higher risk than chicks and adults, P = 0.02) and larger flock size. The latter could represent an indirect effect because larger flock size was also associated with a mixed-species flock structure, but a larger flock could speculatively also consist of chickens from more numerous sources compared to small flocks.

Most of the respondents had added new birds to their flock within a year before the outbreak (82%, Table 3) of which many had introduced live birds rather than hatching eggs. Bird-to-bird transmission from diseased birds or clinically healthy carriers was therefore assumed to be the main source of infection, but transmission of *M*. *gallisepticum* through hatching eggs was also a possibility [27]. Owners of SCF may be unaware of the risks of acquiring birds of unknown health status from other farms, live poultry markets or poultry shows and introducing them into their own flock. Moreover, quarantining new chickens for a short period of time may not be sufficient due to the silent carrier state and the fact that respiratory signs may be intermittent. In case of ILTV, virus excretion may be reactivated as a result of mixing birds of different origins or start of lay [28] which means that transmission may not happen soon due to a latency stage. Moreover, more than 30% of the 23 respondents who had owned their flock more than three years before respiratory signs appeared had not acquired new birds during the preceding 12 months of the disease event. Indirect transmission, a long-term carrier state within the flock or in case of *M*. *gallisepticum*, a wildlife source could not be completely ruled out.

Another interesting finding in this study was that despite the serious clinical signs reported, less than ten percent of the flocks had received therapy to alleviate the respiratory infection (Table 4). Over-the-counter products were mostly used to alleviate clinical signs. Further, and in accordance with national recommendations to limit the use of antimicrobials (Swedish Medical Products Agency, 2019), none of the respondents reported having used antimicrobial drugs. Mild respiratory infections in SCF will usually not require antimicrobial treatment, unless complicated by secondary bacterial involvement. Further, no drugs are available that will alleviate clinical signs in birds affected by ILTV alone [28]. In this study, full recovery was reported by less than 40% of the flock owners, which could be explained by the severity of the outbreaks, the high occurrence of coinfections, and the long-term clinical signs *M*. *gallisepticum* and *A. paragallinarum* sometimes cause. Similarly, none of the respondents in this study reported having used vaccines to prevent respiratory infections or other poultry diseases. This agrees with earlier findings from Finland and Sweden in which less than two percent of SCF owners had used vaccines [9, 11]. Vaccination of chickens in small flocks, mostly against Marek’s disease, appear to be somewhat more common in some other countries such as the United Kingdom, Canada and USA [1, 25, 29].

In accordance with a previous study [1] the respondents in this study often gained information and advice on animal health from other owners and non-professionals (39.9%, Table 4). Notably, less than 35% or the respondents had been in contact with a veterinarian who was not affiliated to the diagnostic laboratory. Contacts with veterinarians at the diagnostic laboratory (SVA poultry veterinarians) could be assumed to mainly involve questions concerning diagnostics, but they may also have included general information on preventive measures and outbreak management. It was however not clear whether the other veterinary contacts involved practitioners and if they had been consulted prior to after having obtained a laboratory-confirmed diagnosis. A previous report from USA reported that only 42.7% of small-scale and backyard poultry and livestock owners sought veterinary help despite experiencing an animal health concern [5]. The reasons for this may be many and are poorly understood which warrants further study.

Even though cleaning and disinfection routines following the outbreaks (Table 4) were not associated with clinical recovery, the results showed that there is a potential for improvement. Earlier studies have reported similar findings and also suggested that the biosecurity routines on small non-commercial chicken farms may be inadequate [8, 9]. This is an area where veterinary practitioners potentially could offer more help. From this study it appears that there is an urgent need for improved communication between veterinarians and owners of small poultry flocks. Veterinary services should focus on recommendations regarding disease control and prevention, including biosecurity measures and vaccination, in addition to veterinary care when outbreaks occur. Moreover, commercial poultry farmers should limit direct and indirect contacts when selling chickens to small hobby flocks to avoid any exchange of respiratory pathogens.

## Conclusions

This study showed that outbreaks of *A. paragallinarum*, *M*. *gallisepticum* and ILTV are geographically widespread in SCF in Sweden and that the consequences of such outbreaks in SCF are many and appear difficult for the flock owners to handle. In this study, mixed respiratory infection was common and was associated with morbidity and mortality and many owners decided to euthanize some chickens or the entire flock. A tenth of owners ceased keeping poultry following an outbreak, which further stresses the severe consequences of respiratory infections. Full clinical recovery was reported in less than 40% of the examined flocks indicating not only acute but also long-term chronic health effects in the majority of the infected flocks. The severity of the outbreaks and the poor clinical outcome in this study call for improved veterinary care and preventive measures in small poultry flocks.

### Supplementary Information

**Additional file 1**. Questionnaire (translated from Swedish) (Q = question number).

## Data Availability

The datasets used and/or analysed in this study are available from the first author on reasonable request following the terms defined by the National Veterinary Institute in Sweden.

## Abbreviations

*A*. * paragallinarum*: *Avibacterium* *paragallinarum*
ILTV: Infectious laryngotracheitis virus
*M*. *gallisepticum*: *Mycoplasma* *gallisepticum*
SCF: Small chicken flocks
