# Supplementary material for: Questionnaire study suggests grave consequences of infectious laryngotracheitis, infectious coryza and mycoplasmosis in small chicken flocks
Source: Acta Vet Scand. 2023 Sep 14;65:39. doi: 10.1186/s13028-023-00703-z (PMC10500715; doi:10.1186/s13028-023-00703-z)
Supplement: Supplementary file 1 — Additional file 1. Questionnaire (translated from Swedish) (Q = question number). [file 13028_2023_703_MOESM1_ESM.pdf]

Additional file 1; Questionnaire (translated from Swedish) (Q=question number)

Part 1. General background

Q1. Participation code[a]

____

[a]Provided by the National Veterinary Institute (SVA) and should be entered by respondent.

Q2. Postal code

____

Q3. How many chickens (including chicks) do you currently keep in your flock?

- □ 0
- □ 1–10
- □ 11–50
- □ 51–100
- □ 101–250
- □ 251–500
- □ 501 or more

Q4. Which categories of chickens do you keep in your flock? (Multiple choices possible.[b])

- □ Mixed/crossed breeds
- □ Pure-bred
- □ Heritage breeds
- □ Layer hybrids
- □ Broiler hybrids
- □ Other

[b]This question was only visible to those who replied they currently kept one or more chickens (Q3).

Q5. Do you also keep other poultry than chickens (within the same flock or as a separate flock(s)? (Multiple choices possible.[b])

- □ No
- □ Yes, turkeys
- □ Yes, domestic ducks, Muscovy ducks and/or domestic geese
- □ Yes, one or more other poultry species (e.g., quails, guinea fowl, peacocks, pheasants)

[b]This question was only visible to those who replied they currently kept one or more chickens (Q3).

Part 2. Flock status prior to the outbreak

Q6. How long had you kept poultry before the onset of respiratory symptoms?

- □ Less than 6 months
- □ More than 6 months–up to 12 months
- □ Between 1–3 years
- □ More than 3 years
- □ I don´t know

Q7. Were your chickens vaccinated against any disease within 12 months before onset of respiratory symptoms?

- □ Yes
- □ No
- □ I don´t know

Q8. What were your chickens vaccinated against? (Multiple choices possible.[c])

- □ Infectious laryngotracheitis (ILT)
- □ Infectious bronchitis (IB)
- □ Marek's disease
- □ Coccidiosis
- □ I don´t know
- □ Other

[c]*This question was only visible to those who replied yes (Q7).*

Q9. Were new chickens or hatching eggs introduced to the flock prior to the respiratory outbreak?

- □ No
- □ Yes, within the recent month
- □ Yes, within 1–5 months
- □ Yes, within 6–12 months
- □ I don´t recall

Q10. What did you acquire?[d]

- □ I only acquired live birds
- □ I acquired both hatching eggs and live birds
- □ I only acquired hatching eggs

[d]*This question was only visible to those who replied yes (Q9).*

Q11. From which sources do you acquire new chickens? (Multiple choices possible.[b])

- □ I do not acquire live birds or hatching eggs from other farms
- □ From other small chicken flocks
- □ From commercial flocks
- □ From live bird markets/poultry shows
- □ From abroad (hatching eggs/live birds)

[b]*This question was only visible to those who replied they currently kept one or more chickens (Q3)*

Q12. Which country did you import hatching eggs/chickens from?

____

Part 3. Clinical course of the respiratory outbreak

Q13. When did the chickens show the first symptoms of respiratory disease?

- □ Jan–Mar
- □ Apr–Jun
- □ Jul–Sep

□ Oct–Dec
□ I don´t know

Q14. Which age groups showed symptoms of disease? Multiple choices possible.
□ Chicks (0–6 weeks)
□ Young birds (7 weeks–5 months)
□ Adults (older than 5 months)
□ I don´t know

Q15. Which of the following symptoms were observed? (Multiple choices possible.)
□ General disease signs (weakness/lethargy, less active, ruffled plumage)
□ Reduced appetite
□ Sneezing, coughing
□ Abnormal breathing sounds (rales, wheezing)
□ Blood or other discharge/secretions (on/in the beak, on feathers or in the hen house)
□ Swelling around the eyes or swelling of the head
□ Eye discharge
□ Decreased egg production
□ Mortality

Q16. What was the approximate flock mortality level due to respiratory disease?[e]
□ 1–20%
□ 21–50%
□ 51–80%
□ 81–99%
□ All (100%)
□ I don´t recall

[e]*This question was only visible to those who replied they had observed mortality (Q15)*

Q17. What was the approximate level of euthanasia due to respiratory disease?
□ None
□ 1–20%
□ 21–50%
□ 51–80%
□ 81–99%
□ All (100%)
□ I don´t recall

Section 4. Outbreak management

Q18. Who did you consult regarding the respiratory outbreak? (Multiple choices possible.)
□ Other poultry owners or non-professionals
□ Poultry veterinarians at the National Veterinary Institute (SVA)
□ Other veterinarians

Q19. Did the birds receive any drugs or other products due to the outbreak?

□ No birds were treated

□ Some birds were treated

□ All birds were treated

Q20. Which drugs/products were used? Please enter the name of the drug(s)/product(s).

____

Section 5. Post-outbreak management

Q21. Did the flock recover?[f]

□ Yes

□ No

[f]This question was visible to those who replied less than 100% mortality and/or euthanasia or replied lack of recollection (Q16 & Q17).

Q22. What happened to the chicken flock after the outbreak?

□ All birds were replaced

□ Some new birds were added to the flock

□ I kept my birds, but I didn’t acquire more

□ I ceased keeping chickens

Q23. Was cleaning and disinfection carried out after the outbreak? (Multiple choices possible.[g])

□ Neither cleaning of the chicken coop or surrounding environment was carried out

□ Manure was removed

□ Soaking with water

□ Washing (using a scrub, brush or equivalent)

□ High pressure cleaning

□ Disinfection

[g]This question was visible to all except those who ceased keeping chickens (Q22).

Q24. Free text field for further short comments or views

____
